# 2204. Pathogen Co-infections and Trends in Influenza-like Illness in PAIVED

**DOI:** 10.1093/ofid/ofac492.1823

**Published:** 2022-12-15

**Authors:** Stephanie A Richard, Christina Schofield, Limone Collins, Christina Spooner, Srihari Seshadri, Anuradha Ganesan, Wesley R Campbell, David Hrncir, Tahaniyat Lalani, Tyler Warkentien, Katrin Mende, Ana E Markelz, Catherine M Berjohn, Bruce McClenathan, Jitendrakumar Modi, Alan Williams, Timothy Burgess, Rhonda E Colombo

**Affiliations:** Infectious Disease Clinical Research Program, Department of Preventive Medicine and Biostatistics, Uniformed Services University of the Health Sciences, Bethesda, MD, USA, Bethesda, MD; Madigan Army Medical Center Division of Infectious Diseases, Infectious Disease Clinical Research Program, Tacoma, Washington; Immunization Healthcare Division, Defense Health Agency, Bethesda, Maryland; Immunization Healthcare Division, Defense Health Agency, Bethesda, Maryland; Immunization Healthcare Division, Defense Health Agency, Bethesda, Maryland; Infectious Disease Clinical Research Program, Department of Preventive Medicine and Biostatistics, Uniformed Services University of the Health Sciences, Bethesda, MD, USA, Walter Reed National Military Medical Center, Bethesda, Maryland; Walter Reed National Military Medical Center, Bethesda, Maryland; Carl R. Darnall Army Medical Center/Wilford Hall Ambulatory Surgical Center, Fort Hood, Texas; Naval Medical Center Portsmouth, Portsmouth, Virginia; Naval Medical Center Portsmouth, Portsmouth, Virginia; Infectious Disease Clinical Research Program, Department of Preventive Medicine and Biostatistics, Uniformed Services University of the Health Sciences, Bethesda, MD, USA, Bethesda, MD; Brooke Army Medical Center, San Antonio, Texas; Naval Medical Center San Diego Division of Infectious Diseases, Infectious Disease Clinical Research Program, San Diego, CA; Womack Army Medical Center, Fort Bragg, North Carolina; Naval Health Clinic, Annapolis, MD, Anapolis, Maryland; Uniformed Services University of the Health Sciences, Bethesda, Maryland; Infectious Disease Clinical Research Program, Department of Preventive Medicine and Biostatistics, Uniformed Services University of the Health Sciences, Bethesda, MD, USA, Bethesda, MD; Infectious Disease Clinical Research Program, Department of Preventive Medicine and Biostatistics, Uniformed Services University of the Health Sciences, Bethesda, MD, USA, The Henry M. Jackson Foundation for the Advancement of Military Medicine, Madigan Army Medical Center Division of Infectious Diseases, Tacoma, Washington

## Abstract

**Background:**

The Pragmatic Assessment of Influenza Vaccine Effectiveness in the Department of Defense (DoD) (PAIVED) is a multicenter, multiservice study assessing influenza vaccine effectiveness in active-duty service members, retirees, and dependents. In its fourth season (2021/22), PAIVED offers a unique opportunity to examine influenza-like illness (ILI) trends prior to and during the COVID-19 pandemic in a prospectively followed, well-defined cohort.

**Methods:**

Over the past 4 influenza seasons, PAIVED has enrolled DoD beneficiaries who were randomized to receive egg-based, cell-based, or recombinant-derived influenza vaccine. Participants provided some basic demographic information and were then sent a weekly text or email that inquired about ILI symptoms, defined as 1) having cough or sore throat, plus 2) feeling feverish/having chills or having body aches/fatigue. Participants with ILI completed a daily symptom diary for one week and submitted a nasal swab for PCR-based pathogen detection.

**Table 1.**
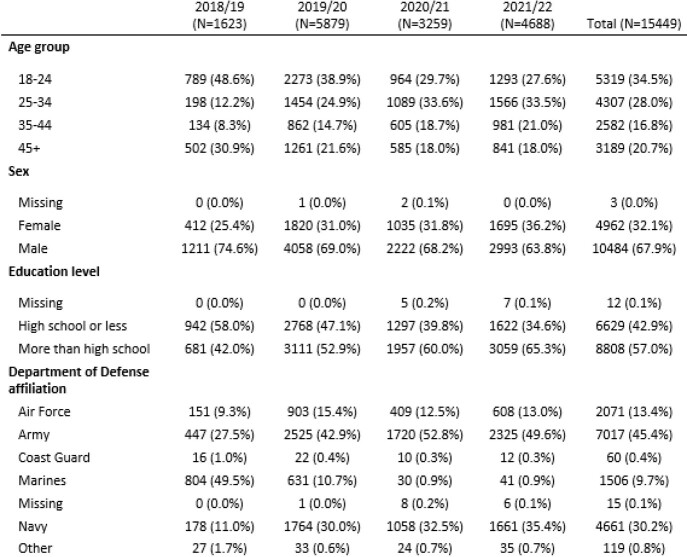
Demographic characteristics of PAIVED participants over four seasons

**Figure 1.**
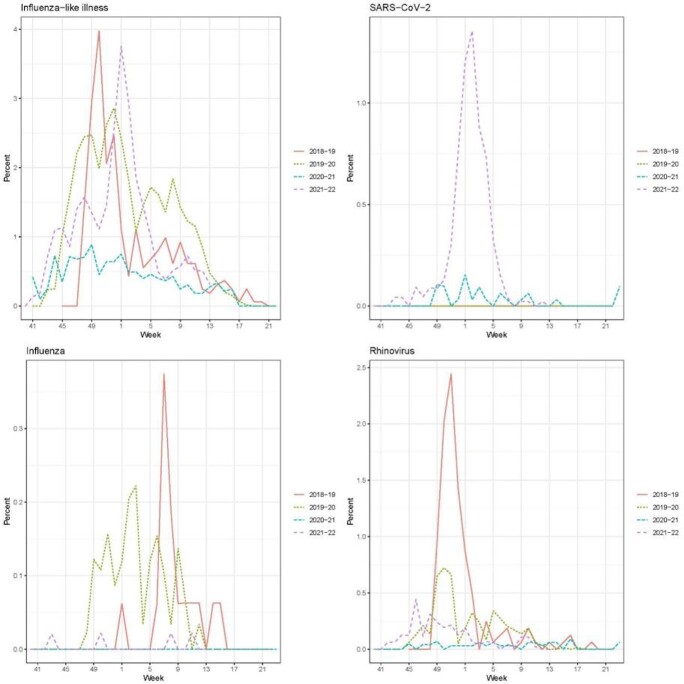
Percent of PAIVED participants with influenza-like illness, SARS-CoV-2, influenza, and rhinovirus identified in swab samples collected over four seasons.

**Results:**

Over the 4 seasons, 15,449 participants were followed for ILI (Table 1) with 3,407 participants reporting a total of 3,985 ILIs. For the 2021/22 season, ILI reports peaked in January (Figure 1). Overall, 4.7% of episodes had more than one pathogen identified (Table 2). Among the 122 coinfections identified to date, most were coinfections with rhinoviruses (91/122, 75%), including rhinovirus coinfections with seasonal coronaviruses (29, 24%), metapneumovirus (18, 15%), SARS-CoV-2 (17, 14%), and influenza (14, 11%). SARS-CoV-2 and influenza were found together in one sample. The lab data will continue to be processed for the current season (2021/22).

**Table 2.**
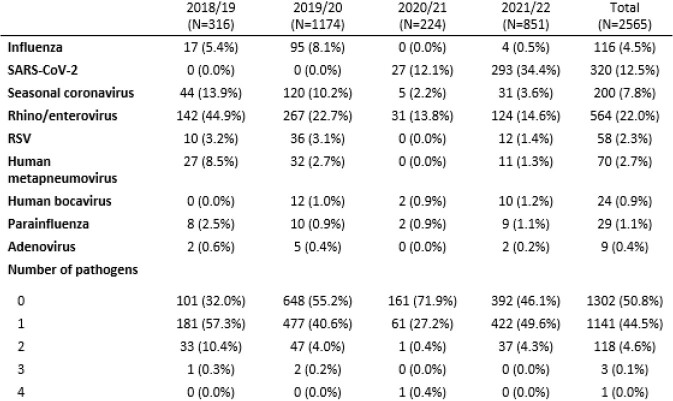
Pathogens identified in PAIVED nasal swabs over four seasons

**Conclusion:**

ILI rates were lowest during the third year (2020/21), consistent with national influenza surveillance reports of influenza and outpatient ILI activity, suggesting that measures taken to reduce transmission of SARS-CoV-2 reduced the spread of other respiratory viruses. The emergence of the SARS-CoV-2 omicron variant in December 2021 was associated with higher ILI rates. Among those individuals for whom a sample was collected, coinfections were highest in 2018/19. Data collection and specimen analysis are ongoing for 2021/22.

**Disclosures:**

**Jitendrakumar Modi, MD**, GlaxoSmithKline: I am a paid speaker for GSK. I do not speak for their flu brand. **Timothy Burgess, MD, MPH**, AstraZeneca: The HJF, in support of the USU IDCRP, was funded to conduct or augment unrelated Phase III Mab and vaccine trials as part of US Govt. COVID19 response.

